# Metabolites of Siberian Raspberries: LC-MS Profile, Seasonal Variation, Antioxidant Activity and, Thermal Stability of *Rubus matsumuranus* Phenolome

**DOI:** 10.3390/plants10112317

**Published:** 2021-10-27

**Authors:** Nina I. Kashchenko, Daniil N. Olennikov, Nadezhda K. Chirikova

**Affiliations:** 1 Laboratory of Medical and Biological Research, Institute of General and Experimental Biology, Siberian Division, Russian Academy of Science, 670047 Ulan-Ude, Russia; olennikovdn@mail.ru; 2 Department of Biology, Institute of Natural Sciences, North-Eastern Federal University, 677027 Yakutsk, Russia; hofnung@mail.ru

**Keywords:** *Rubus matsumuranus*, Rosaceae, phenolic compounds, ellagitannins, high-performance liquid chromatography, mass spectrometry, antioxidant activity, seasonal variation

## Abstract

*Rubus matsumuranus* H. Lev. & Vaniot, a famous Siberian shrub of the Rosaceae family, is used in the folk medicine of nomads (Buryats, Yakuts, Soyots, and Mongols) as a remedy for the treatment of diseases of the respiratory and hepatobiliary systems. The lack of scientific information on *R. matsumuranus* leaves contributed to the investigation of the metabolomic profile and biological activity of this plant. In this study, metabolites of *R. matsumuranus* leaves in three stages (active growth, flowering, and fruiting) were characterised using high-performance liquid chromatography with photodiode array and electrospray ionisation triple quadrupole mass spectrometric detection (HPLC-PDA-ESI-tQ-MS). In total, 63 compounds were identified, including gallic acid derivatives, hydroxycinnamates, catechins, procyanidins, flavonols, and ellagitannins. Lambertianin C (57.11 mg/g of dry weight, DW), miquelianin (39.63 mg/g DW), and kaempferol-3-*O*-glucuronide (31.18 mg/g DW) were the major compounds in *R. matsumuranus* leaves. As a result of the HPLC-PDA-based assay to determine the antioxidant activity, it was revealed that lambertianin A, sanguiin H6, lambertianin C, and sanguiin H11 were effective scavengers of free radicals (2,2-diphenyl-1-picrylhydrazyl, DPPH^•^) and possessed Fe^2+^-chelating activity. After an investigation of the phenolic content in infusions and decoctions obtained by extraction with water at different temperatures, it was revealed that a hot infusion (80 °C) is a phenolic-rich preparation of *R. matsumuranus* leaves. Our research suggests that *R. matsumuranus* leaves are a rich source of phenolic compounds with high antioxidant properties and that this could be a prospective plant for new functional products.

## 1. Introduction

An increase in the range of medicines based on plant raw materials is one of the directions of development of the pharmaceutical industry [1]. The investigation and implementation of traditional medicinal plants into practice is a promising area. In addition, plant objects that are systematically close to the official ones and have a sufficient raw material base can be used. Such plants can be a possible source of functional food that has positive physiological benefits beyond their nutritional function [2]. The genus *Rubus* of the Rosaceous family is a potential source of functional products due to the variety of dietary fruits and leaf-based herbal teas [3,4,5,6]. In addition, the European Medicines Agency has approved the use of *Rubus idaeus* leaf infusions and extracts as herbal medicinal products based on their traditional uses [7].

**Figure 1:**
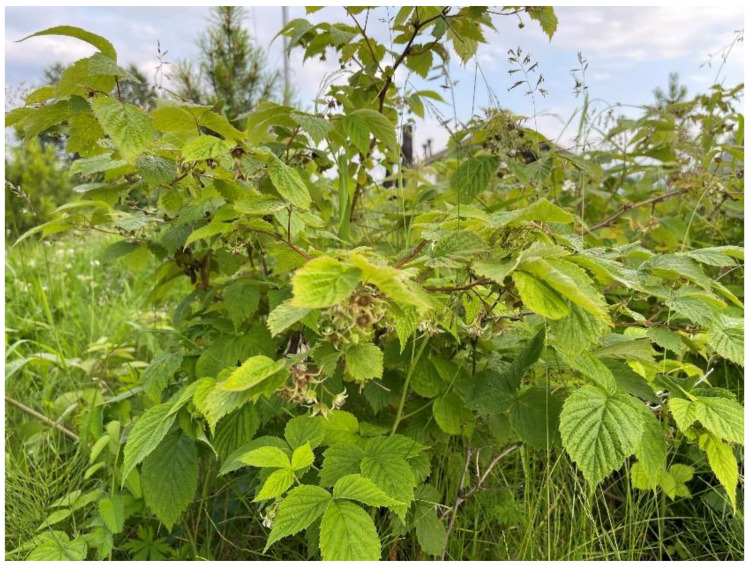
Flowering shoots of *Rubus matsumuranus* in its natural habitat (Republic Buryatia, Ivolginsky District).

The traditional medicine of the nomadic peoples of Siberia (Buryats, Yakuts, Soyots, and Mongols) was influenced by almost complete geographical isolation from the centers of civilization [8]. Nomads, in the interests of self-preservation, developed their own folk medicine, in which plants of the local flora were used as medicinal preparations [9]. *Rubus matsumuranus* H. Lev. & Vaniot (*Rubus sachalinensis* var. *sachalinensis*) is one of the plants of traditional medicine of Siberian nomads (Figure 1). Botanically, *R. matsumuranus* is a shrub 30–100 cm tall with shoots covered with yellowish or brown needles with an admixture of stalked glands. The leaves are trifoliate, oblong-ovate or broadly lanceolate, and long or shortly pointed. Fruits are red drupes. It grows in plain and mountain forests in clearings, stony placers, and in subarctic woodlands in Western and Eastern Siberia, Northern Mongolia, and the Far East [10]. According to ethnopharmacological data, the Yakut nomads call this type of raspberries биэ эмиийэ. In Yakut folk medicine, a decoction of leaves was used for jaundice and kidney disease [11]. Mongolian and Buryat nomads used a decoction of young *R. matsumuranus* shoots (бoopoлзгoнo, зaбaн зэдэгэнэ) as a remedy for diseases of the respiratory system and the treatment of pneumonia [12].

There are no current scientific data regarding the metabolites of *R. matsumuranus*. However, there is some information about the chemical composition of a close species, *R. sachalinensis*. Therefore, the flavonols quercetin and kaempferol, as well as their glucuronides [13] and polysaccharides [14], have been identified in *R. sachalinensis*. Additionally, the good antioxidant activity of this plant species was reported [15]. Due to their high antioxidant activity, species of the *Rubus* genus are used for cosmetic purposes [16]. Analysing information on chemical studies of other species of the genus *Rubus*, an increased interest in phenolic compounds was revealed, which was explained by their high biological activity [17,18]. *Rubus* phenols are a diverse group of compounds, including phenolic acids, flavonoids, gallotannins, and ellagitannins [19,20]. Ellagitannins, as marker compounds of the Rosaceae family, are of particular interest and represent a complex class of polyphenols consisting of units of hexahydroxydiphenoyl fragments as well as ellagic and gallic acids, and esterified with a carbohydrate, usually glucose [21]. Ellagitannins have various biological activities, including antioxidant [22], anti-inflammatory [23], and antiproliferative [24] activities, and are prospective compounds for investigation.

This report aimed to estimate the prospects of *R. matsumuranus* leaves as a possible functional product. Thus, the first detailed metabolomics profiling was realised for *R. matsumuranus* leaf extract in three stages (active growth, flowering, and fruiting) using HPLC-PDA-ESI-tQ-MS/MS (high-performance liquid chromatography with photodiode array and electrospray ionisation triple quadrupole mass spectrometric detection). Considering that most metabolites found in the *Rubus* genus were phenolics, the antioxidant potential of *R. matsumuranus* leaf extract was studied using an HPLC-PDA-based antioxidant activity assay to track the active components. To assess the possible use of herbal tea from *R. matsumuranus* leaves, the stability and content of phenolic compounds were analysed in infusions and decoctions. To the best of our knowledge, this is the first study of *R. matsumuranus* leaf metabolites.

## 2. Results and Discussion

### 2.1. Metabolites of Rubus matsumuranus Leaves: LC-MS Profile

**Figure 2:**
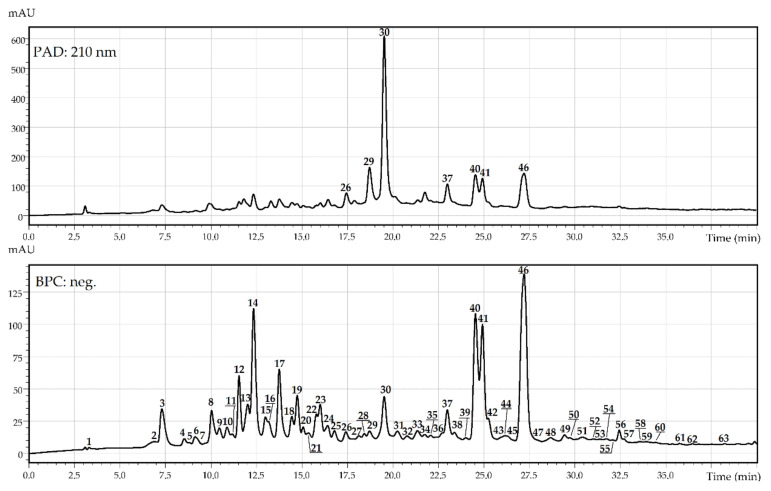
High-performance liquid chromatography with photodiode array (PAD: 210 nm) and electrospray ionization triple quadrupole mass spectrometric detection in base peak chromatogram mode (BPC, negative ionization) of *R. matsumuranus* leaves. Compounds are numbered as listed in Table 1.

The chromatographic profile of *R. matsumuranus* leaves was performed by high-performance liquid chromatography with photodiode array and electrospray ionisation triple quadrupole mass spectrometric detection (HPLC-PDA-ESI-tQ-MS). Components of *R. matsumuranus* leaves were identified after processing the retention times and spectral data. The data obtained were compared with standard substances and literature data. At the preliminary stage of the experiment, the extraction conditions were selected for *R. matsumuranus* leaves (Table S1). Thus, the selection of solvents (methanol, ethanol, isopropanol, and water) was implemented, in addition to the determination of the solvent–material ratio and temperature regime (10–90 °C). Ultrasonic and water bath-assisted methods of extraction were studied. The final extraction conditions were 100% methanol with a solvent–material ratio of 10:1 and sonication (30 min, 50 °C). After comparison of *R. matsumuranus* leaves from three seasonal harvests (active growth, flowering, and fruiting), the HPLC-PDA-ESI-tQ-MS chromatogram of leaves collected during flowering demonstrated the maximal content of biological compounds with interpretable data (Figure 2), the details of which are given in Table 1.

#### 2.1.1. Gallic Acid Derivatives

Gallic acid (**4**) and three gallic acid glycosides (**1**, **5**, **7**, **16**, **23**, **24**) were found in *R. matsumuranus* leaves. Compounds **1** and **7** were detected as *O*-galloyl-dihexose and *O*-galloylhexose, respectively, due to the negative mass spectrum showing deprotonated ions [M-H]^−^ with *m*/*z* 493 and 331, respectively, and the fragmentation patterns corresponded to the loss of gallic acid and hexose units [30]. Compound **24** showed a deprotonated ion with *m*/*z* 635 and a variety of daughter ions with *m*/*z* 483 and 331 (loss of galloyl units), *m*/*z* 169 (deprotonated gallic acid), and *m*/*z* 125 (trihydroxyphenol moiety) and was identified as tri-*O*-galloyl-hexose [31]. Gallic acid (**4**) was previously found in *R. fruticosus* [32] and *R. idaeus* leaves [33].

#### 2.1.2. Hydroxycinnamates

1-*O*-Caffeoylquinic acid (**2**), 3-*O*-caffeoylquinic acid (**13**), *O*-caffeoyl-hexose (**14**), 5-*O*-caffeoylquinic acid (**17**), 4-*O*-caffeoylquinic acid (**18**), and 5-*O*-feruloylquinic acid (**19**) were detected in *R. matsumuranus* leaves. Hydroxycinnamates are common components of the genus *Rubus* and have been found in many species. Moreover, 3-*O*-caffeoylquinic acid (**13**) and 5-*O*-caffeoylquinic acid (**17**) were revealed in *R. ulmifolius* leaves [34], *R. glivicensis*, *R. fasciculatus*, *R. radula*, and *R. montanus*, among others [35].

#### 2.1.3. Catechins and Procyanidins

Procyanidins B_1_ (**9**) and B_2_ (**11**), gallocatechin (**8**), catechin (**10**), epicatechin (**12**), and catechin-*O*-gallate (**31**) were revealed in *R. matsumuranus* leaves. Catechin (**10**) was detected earlier in the aerial part of *R. coriifolius* [36] and in the leaves of *R. fruticosus* and *R. idaeus* [37]. Epicatechin (**12**) was detected in the aerial part of *R. coriifolius* [36] and the leaves of *R. fruticosus* and *R. idaeus* [37]. Procyanidins B_1_ (**9**) and B_2_ (**11**) were found in *R. idaeus* shoots and *R. fruticosus* leaves [37,38,39].

#### 2.1.4. Flavonols

In *R. matsumuranus* leaves, 21 compounds were determined to be flavonols in glycoside form. By comparing their mass spectral data with those reported previously [25,26,27,28,29] and with reference standards, these flavonols were identified as derivatives of quercetin (12 compounds) and kaempferol (9 compounds). The quercetin group of flavonols was the largest with non-acylated and acylated fragments linked with carbohydrate fragments. There were non-acylated derivatives of quercetin, such as quercetin-3-*O*-rutinoside (**38**; rutin), quercetin-3-*O*-glucoside (**39**; isoquercitrin), and quercetin-3-*O*-glucuronide (**40**; miquelianin). Unknown compounds were acylated derivatives of quercetin *O*-hexuronides, providing the same MS/MS moieties as *m*/*z* 477 (quercetin *O*-hexuronide) and 301 (quercetin). Notably, acylated quercetin glycosides had fragments of *O*-malonyl, *O*-acetyl, and hexuronic acid in various ratios, such as 1:0:1 (quercetin *O*-(*O*-malonyl)-hexuronide, **42**), 0:1:1 (quercetin *O*-(*O*-acetyl)-hexuronide, **44**), 1:1:1 (quercetin *O*-(*O*-acetyl-*O*-malonyl)-hexuronide, **46, 47**), 2:1:1 (quercetin *O*-(*O*-acetyl-di-*O*-malonyl)-hexuronide, **50**), 1:2:1 (quercetin *O*-(di-*O*-acetyl-*O*-malonyl)-hexuronide, **52**), 0:3:1 (quercetin *O*-(tri-*O*-acetyl)-hexuronide, **56, 57**), and 1:3:1 (quercetin *O*-(tri-*O*-acetyl-*O*-malonyl)-hexuronide, **58**).

Fewer compounds were kaempferol derivatives. Only kaempferol-3-*O*-glucuronide (**41**) was identified by comparison of *t*_R_, UV, and mass spectrometric data with the reference substance. Other kaempferol derivatives were represented by *O*-acylated *O*-hexuronides. MS patterns of these compounds demonstrated the loss of fragments with *m*/*z* 42 and 86 that corresponded to acetyl and malonyl fragments, respectively. Different combinations of acylated kaempferol *O*-hexuronides were revealed, in particular malonyl (kaempferol *O*-(*O*-malonyl)-hexuronide, **43**); acetyl (kaempferol *O*-(*O*-acetyl)-hexuronide, **45**); acetyl/malonyl (kaempferol *O*-(*O*-acetyl-*O*-malonyl)-hexuronide, **48, 49**); acetyl-di-*O*-malonyl (kaempferol *O*-(*O*-acetyl-di-*O*-malonyl)-hexuronide, **51**); di-acetyl/malonyl (kaempferol *O*-(di-*O*-acetyl-*O*-malonyl)-hexuronide, **53**); tri-acetyl (kaempferol *O*-(tri-*O*-acetyl)-hexuronide, **59**); and tri-acetyl/malonyl (kaempferol *O*-(tri-*O*-acetyl-*O*-malonyl)-hexuronide, **60**).

Quercetin and kaempferol and their derivatives are the most common flavonoids in the *Rubus* genus. Both flavonols were detected previously in *R. ulmifolius* [40], *R. idaeus*, *R. saxatilis*, *R. fruticosus*, *R. occidentalis*, *R. odoratus*, *R. caesius* [41], and *R. erythrocladus* [42]. Quercetin-3-*O*-glucoside (**39**) and kaempferol-3-*O*-glucuronide (**41**) were isolated from *R. idaeus* leaves [43].

#### 2.1.5. Ellagic Acid Derivatives and Ellagitannins

Ellagic acid (**37**), 9 ellagic acid glycosides (**32**, **34**–**36**, **54**, **55**, **61**–**63**), and 12 ellagitannins (**6**, **15**, **20**–**22**, **25**–**30**, **33**) were revealed in *R. matsumuranus* leaves. The following ellagic acid glycosides were identified: ellagic acid *O*-pentoside-*O*-hexoside (**32**), ellagic acid *O*-hexoside (**34**, **35**), ellagic acid *O*-pentoside (**36**), ellagic acid *O*-methyl ester *O*-pentoside (**54**, **55**), ellagic acid *O*-di-methyl ester (**61**, **62**), and ellagic acid *O*-tri-methyl ester (**63**). The identification of ellagic acid glycosides was carried out based on the presence of ions with *m*/*z* 301, specific for ellagic acid derivatives, as well as the loss of fragments with *m*/*z* 14 (methyl), 132 (pentose), and 162 (hexose).

Ellagitannins of different structural types, such as hexahydroxydiphenoyl glucose (pedunculagin (**6**, **27** as isomers)), hexahydroxydiphenoyl-galloyl-glucose (tellimagrandins I_1_ (**15**), I_2_ (**20**), II_1_ (**21**), II_2_ (**28**), potentillin (**22**)), and ellagitannins with a sanguisorboyl unit (sanguiins H10 (**25**), H6 (**29**), H11 (**33**), and lambertianins A (**26**) and C (**30**)) were found using the reference standards and literature data. Compound **27** was identified as a possible isomer of pedunculagin (**6**) due to the presence of typical ions of deprotonated fragments [M-H]^−^ with *m*/*z* 783, 481 (loss of hexahydroxydiphenoyl [HHDP] group), and 301 (loss of HHDP-glucose) [44,45].

Discovered ellagitannins were previously found in the genus *Rubus*. Pedunculagin (**6**) was detected previously in *R. caesius* leaves [46]. Tellimagrandin I (**15**) was found in the leaves of *R. fruticosus*, and tellimagrandin II (**21**) was revealed in the leaves of *R. hiraseanus*, *R. hirsutus*, and *R*. × *masakii* [47]. Another ellagitannin of the hexahydroxydiphenoyl-galloyl-glucose type, potentillin (**22**), was detected previously in *R. arcticus* leaves [48] and *R. idaeus* shoots [38]. Okuda et al. [47] suggested using the compounds sanguiin H6 (**29**) and sanguiim H11 (**33**) as chemotaxonomic markers of the genus *Rubus*. This statement applies to the considered plant object. In addition, these compounds were found in many species of the genus *Rubus*, for example, *R. coreanus*, *R. crataegifolius*, *R. fruticosus*, *R. hirsutus*, *R. parvifolius*, and *R. palmatus*. Later, Tanaka et al. demonstrated the fallacy of the idea of the presence of sanguiin H11 (**33**) as a chemotaxonomic compound of the genus *Rubus* by isolating the tetramer lambertianin D from *R. lambertianus*, which is an isomer of sanguiin H11 (**33**) [49]. Thus, chemotaxonomic compounds of the genus *Rubus* include not only sanguiin H6 (**29**) but also lambertianin C (**30**) and D.

### 2.2. Quantitative Content and Seasonal Phenolic Profile of R. matsumuranus Leaves

Possible trends in the phenolic constituent pattern of *R. matsumuranus* leaves were investigated during various growth periods. The samples were collected during the active growth (May), flowering (July), and fruiting (September) phases. When quantitatively analysing the derivatives of gallic acid in seasonal samples of *R. matsumuranus* leaves, it was found that this group of compounds was present in all phases. A gradual decrease in gallic acid (**4**) content was observed from May to September, from 2.03 to 0.24 mg/g. A possible explanation for this phenomenon is the fact that gallic acid is a precursor compound of the biosynthesis of hydrolysable tannins [50,51]. A similar trend with the maximum content of compounds in May was noted for the derivatives of gallic acid—galloyl-hexoses (**1**, **16**, **23**, **24**). According to a probable assumption, ellagitannins and gallotannins were derived from galloylglucoses by the addition of complementary galloyl residues or by oxidation [52]. Biosynthesis of the precursor glucogallin (**5**) was formed during the esterification of gallic acid and glucose, followed by the formation of di-, tri-, tetra-, and pentagalloylglucose during the re-esterification reaction [53]. Then, the transformation of gallotannins into ellagitannins occurred by oxidative binding of galloyl groups [54]. A similar profile was noted for both herbaceous (*Geranium sylvaticum*) and woody (*Quercus robur*) plant species [55,56].

The content of all caffeoylquinic acids, as well as 5-*O*-feruloylquinic acid, decreased in September. The maximal content of all hydroxycinnamates was noted in May samples. Thus, the difference between *O*-caffeoyl-hexose (**14**) collected in May differed from the samples collected in September by more than 3-fold. The content of 3-*O*-caffeoylquinic acid (**13**) in spring samples was 1.92-times higher than that in autumn samples. The same trend was noted for 5-*O*-feruloylquinic acid (**19**)—3.86 mg/g in May samples vs. 1.98 mg/g in the September samples. According to the literature, such an increase in the content of hydroxycinnamates in leaves at the beginning of the growing season is typical for many plant objects, for example, *Juglans regia* [57], *Ribes nigrum* [58], and *Sorbus domestica* [59]. A possible reason for this phenomenon is the role of hydroxycinnamates as a precursor of the biosynthesis of polyphenolic compounds, for example, derivatives of flavan-3-ol, in particular catechins [60,61].

There were various trends in the accumulation of catechins and procyanidins in *R. matsumuranus* leaves collected in different seasons. In the case of gallocatechin (**8**), catechin (**10**), and epicatechin (**12**), the maximum content was observed in July during the flowering phase. Previously, *R. caucasicus* was noted to accumulate catechin in the leaves during the summer months [62]. Additionally, for another representative of the Rosaceae family, *Filipendula glaberrima*, the maximum content of catechin was noted during the flowering period [63]. In turn, the content of procyanidins B_1_ (**9**) and B_2_ (**11**) gradually increased and reached a maximum during the fruiting phase in September. Similar dynamics of the maximum accumulation of procyanidins in late summer and autumn is quite often observed in deciduous trees [64]. Perhaps this is because procyanidins are involved in the protection of a plant from various abiotic (drought, darkening) and biotic (pathogenic microorganisms, insects) factors throughout the entire life cycle [59,65].

The presence of flavonol glycosides was revealed in all seasonal samples of *R. matsumuranus* leaves. Kaempferol derivatives were generally observed in contents less than 0.01 mg/g. Kaempferol-3-*O*-glucuronide (**41**) and kaempferol *O*-(*O*-acetyl-*O*-malonyl)-hexuronides (**48**, **49**) were the exceptions and demonstrated quantifiable amounts with maximal content in July. Quercetin-3-*O*-glucuronide (miquelianin, **40**) was the dominant compound among the quercetin glycosides. Its maximum content was noticed in July (39.63 mg/g), dropping to 31.15 mg/g in September. The same trend was observed for other quantifiable quercetin derivatives—quercetin-3-*O*-rutinoside (rutin, **38**), quercetin-3-*O*-glucoside (isoquercitrin, **39**), quercetin *O*-(*O*-malonyl)-hexuronide (**42**), quercetin *O*-(*O*-acetyl)-hexuronide (**44**), quercetin *O*-(*O*-acetyl-*O*-malonyl)-hexuronide (**46**), and quercetin *O*-(tri-*O*-acetyl)-hexuronides (**56**, **57**). The exception to this trend was quercetin *O*-(tri-*O*-acetyl-*O*-malonyl)-hexuronide (**58**), with a content less than 0.01 mg/g in July and the maximum in August. Some of the reasons for the increase in the content of quercetin and kaempferol derivatives in the leaves of *R. matsumuranus* in July may be the high air temperature and maximum UV radiation in this month. Higher growth temperatures increased the content of flavonols in strawberries [66]. Flavonoids have been shown to have the ability to reduce photooxidative damage by directly absorbing UV radiation [67]. UV light increases the biosynthesis of flavonoids with the catechol group in skeletal ring B (for example, quercetin derivatives). The latter are better antioxidants than methoxylated flavonoids or flavonoids, with one substitution in the B ring [68,69,70].

When analyzing the ellagitannins of *R. matsumuranus* leaves, the following tendency (with some exceptions) was observed: maximum content during flowering, followed by a decrease in the fruiting phase. This was especially noticeable in relation to the dominant ellagitannins—lambertianin C (**30**) (from 57.11 to 48.10 mg/g), sanguiin H6 (**29**) (from 19.62 to 15.32 mg/g), and lambertianin A (**26**) (from 5.21 to 4.63 mg/g). The results obtained with the maximal content in the warm season correlated with the data of Remberg et al., who found an increase in the content of the main ellagitannins of *R. idaeus* (sanguiin H6 and lambertianin C) at the maximum growing temperature (24 °C) [71]. For tellimagrandins I_1_ (**15**), I_2_ (**20**), II_1_ (**21**), and II_2_ (**28**) the maximal concentrations were found in the July samples, dropping sharply in September. In contrast, the maximum content of pedunculagin (**6**, **27** as an isomer) was observed in the autumn samples (0.67 mg/g in July vs. 0.92 mg/g in September). A similar phenomenon was noted for the leaves of *Liquidambar formosana* [72]. According to the biosynthetic pathway of pedunculagin, it can be formed because of oxidative binding between the two galloyl groups in tellimagrandins [73].

The content of ellagic acid (**37**) was highest in September during the fruiting phase (from 6.24 to 11.20 mg/g). As this is the end of the biosynthetic pathway, ellagic acid may be released during the hydrolysis of ellagitannins [51]. Contents of methyl, di-, and trimethyl esters of ellagic acid (**54**, **55**, **61**–**63**) were observed as less than 0.01 mg/g, which makes it impossible to quantify the compounds, depending on the season. In turn, the maximum content of ellagic acid hexosides (**34**, **35**) and pentoside (**36**) were observed during the flowering phase, while amounts less than 0.01 mg/g of these compounds were observed in the remaining phases.

Information about the accumulated biologically active compounds depending on the time of harvest is valuable data for obtaining an adequate yield of the corresponding plant and preparations derived therefrom. The best time to collect *R. matsumuranus* leaves depends on the required target group of the compounds. Thus, to obtain *R. matsumuranus* raw material maximally enriched in the predominant flavonoids and ellagitannins, the optimal harvest time in Siberia is July.

### 2.3. Bioactivity of R. matsumuranus Leaf Extract: Antioxidant Potential

**Figure 3:**
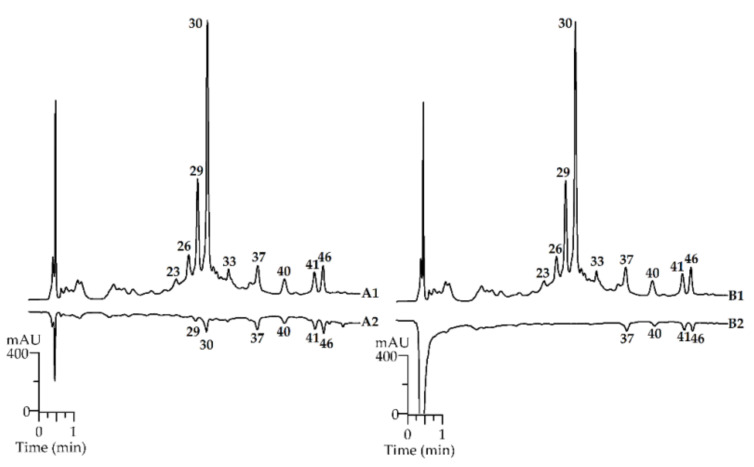
HPLC-PDA-based antioxidant potential of *R. matsumuranus* extract: radical-scavenging assay against DPPH radicals (A1—extract before reaction with DPPH radicals; A2—extract after reaction with DPPH radicals); and Fe^2+^-chelating activity (B1—extract before reaction with Fe^2+^ions; B2—extract after reaction with Fe^2+^ions). Compounds are numbered as listed in Table 1.

It is well known that phenolic compounds have pronounced antioxidant activity [74,75]. It was previously demonstrated that plant species of the *Rubus* genus exhibited high antioxidant activity due to phenolic compounds, especially ellagitannins [76,77]. Study of the antioxidant potential of *R. matsumuranus* extract against DPPH^•^ radicals in a microplate spectrophotometric assay demonstrated the superior scavenging effect with an IC_50_ value of 24.68 ± 0.59 µg/mL; the same parameter for Trolox, which was used as a reference compound, was 9.14 ± 0.16 µg/mL. To further explore the antioxidant potential of the *R. matsumuranus* leaf extract, an HPLC-PDA-based antioxidant activity assay was applied. This method is an effective means for tracking active components [78,79] in the investigated extract and utilises the pre-chromatographic reaction of plant extracts with an excess of free radical 2,2-diphenyl-1-picrylhydrazyl (DPPH^•^) and Fe^2+^ ions (Figure 3). Further comparisons of the chromatograms (untreated and radical-treated samples) allowed for making conclusions about the most active compounds. The obtained two-dimensional chromatogram displayed a high ability for the selected compounds to scavenge radicals or bind transition metal ions in the form of peaks with a reduced area.

Ellagitannins were the most active compounds in *R. matsumuranus* leaf extract, which had clearly reduced peak areas for lambertianin A (peak 26), sanguiin H6 (peak 29), lambertianin C (peak 30), and sanguiin H11 (peak 33) according to the analysis of radical scavenging against DPPH radicals compared to untreated samples. Ellagic acid (peak 37), as well as the flavonoids quercetin-3-*O*-glucuronide (peak 40), kaempferol-3-*O*-glucuronide (peak 41), and quercetin-*O*-(*O*-acetyl-*O*-malonyl)-hexuronide (peak 46) demonstrated less antioxidant activity. These findings support early evidence that an increase in the molecular weight of tannins with a concomitant growth in the number of phenolic hydroxyl groups led to a rise in the scavenging effects of the DPPH radical [80]. The investigation of the Fe^2+^-chelating properties of individual compounds by the ability to bind Fe^2+^ ions revealed the almost complete absence of ellagitannin peaks in the B2 chromatogram and a decrease in ellagic acid and flavonoid peaks. There are various assumptions about the mechanisms of adsorption of transition metal ions with ellagitannins. According to the conclusions of various studies, ion exchange, complexation, chemisorption, and surface adsorption are possible explanations [81]. Thus, the results demonstrated that most phenolic compounds of the *R. matsumuranus* leaf extract engaged in the process of radical scavenging and Fe^2+^-chelating activity, especially the ellagitannins lambertianin A, sanguiin H6, lambertianin C, and sanguiin H11.

### 2.4. Stability of R. matsumuranus Phenolic Compounds in Water Media: Comparison of Infusion and Decoction Composition

Recently, there has been a growing interest in research on the chemical composition of herbal teas. Similar teas are the most popular non-alcoholic beverages in the world due to the large number of health benefits [82,83]. Herbal teas, which are infusions and decoctions from leaves, roots, flowers, and plant seeds, are an important source of phenolic compounds in the human diet [84,85]. To assess the possible use of herbal tea from *R. matsumuranus* leaves, the phenolic compound content was analysed in infusions and decoctions obtained from extraction with water at different temperatures (Table 2).

The maximum content of lambertianines A and C and sanguiins H6 and H11 were recorded for the infusion, which was obtained by the extraction of *R. matsumuranus* leaves with water at 50 °C. The minimum content of the same compounds was observed in the decoction obtained by extraction for 15 min, and with further extraction up to 30 min, amounts less than 0.01 of these ellagitannins were observed. It was previously shown that the content of lambertianin C and sanguiin H6 from *R. idaeus* extract rapidly decreased in a neutral aqueous medium at elevated temperatures (60–80 °C) [86]. There was also an increase in the content of ellagic acid in both decoctions compared to infusions. Previously, a similar effect was described for *R. lambertianus* leaves with prolonged extraction at elevated temperatures [49]. The possible reason for the accumulation of ellagic acid in the decoctions is the fact that ellagitannins are unstable at high temperatures and decompose to ellagic acid [87]. The content of both catechins was maximal in the decoctions. Thus, the maximal gallocatechin concentration was observed for the 15 min decoction, and epicatechin content was noted for the 30 min decoction. The minimum values for both compounds were observed after infusion in cold water. Previously, Zhu et al. [88] reported the stability of green tea catechins upon boiling. Analysis of the hydroxycinnamates in aqueous forms of *R. matsumuranus* leaves revealed that monocaffeoylquinic acids pass better into a boiling infusion (total content 3.97 mg/100 mL) and less pass into a cold infusion (total content 0.97 mg/mL). The maximum content of 5-*O*-caffeoylquinic acid was noted in the boiling infusion, slightly decreasing in the 15 min decoction (1.14 vs. 1.07 mg/100 mL). According to the literature, 5-*O*-caffeoylquinic acid is more stable than 4-*O*-caffeoylquinic acid and undergoes a degradation process upon prolonged heating [89,90]. Flavonols quercetin-3-*O*-glucuronide, quercetin-*O*-(*O*-acetyl-*O*-malonyl)-hexuronide, and kaempferol-3-*O*-glucuronide were quantified in infusions and decoctions of *R. matsumuranus* leaves. The highest levels of quercetin and kaempferol glucuronides were found in the 30 min decoction (8.21 and 8.66 mg/100 mL, respectively). The minimum concentrations of both compounds were found in cold infusions. Quercetin-*O*-(*O*-acetyl-*O*-malonyl)-hexuronide was less stable and its content was highest in the hot infusion, while in the 30 min decoction, its concentration decreased 2.2-fold (9.14 vs. 4.21 mg/100 mL, respectively).

Thus, the hot infusion renders a phenolic-rich aqueous form of *R. matsumuranus* leaves. However, the choice of the aqueous form can vary depending on the goals of the consumer in obtaining a certain class of phenolic compound. Therefore, to obtain the maximum amount of ellagitannins from *R. matsumuranus* leaves, it is recommended that the consumer prepare a warm infusion, while a boiling infusion is suggested for obtaining the maximal content of hydroxycinnamates. For the preparation of aqueous forms enriched with catechins and flavonols, boiling the raw material for 30 min is recommended to obtain the decoction. *R. matsumuranus* herbal tea may be a part of a regular diet as a source of phenolic compounds.

## 3. Materials and Methods

### 3.1. Chemicals

The reference compounds were purchased from ALB (ALB Technology Limited, Mongkok Kowloon HongKong); ChemFaces (Wuhan, China); Sigma-Aldrich (St. Louis, MO, USA); Toronto (Toronto Research Chemicals, North York, ON, Canada) (Table S2). Selected chemicals were from Sigma-Aldrich (St. Louis, MO, USA): acetonitrile for HPLC (Cat. No. 34851, ≥99.9%), 2,2-diphenyl-1-picrylhydrazyl radical (Cat. No. 281689, ≥97%), ferrous sulfate (Cat. No. 1270355), formic acid (Cat. No. F0507, ≥95%); isopropanol (Cat. No. 563935, ≥70%), lithium perchlorate (Cat. No. 205281, ≥95%), methanol (Cat. No. 322415, ≥99.8%), perchloric acid (Cat. No. 244252, ≥70%). Tellimagrandins I and II were isolated previously from *Filipendula ulmaria* [91,92]; lambertianin C was earlier isolated from *Fragaria viridis* [29]. 2-Pyrone-4,6-dicarboxylic acid and potentillin were isolated previously from *Comarum palustre* [93].

### 3.2. Plant Material

*Rubus matsumuranus* leaves were collected from an experimental plantation in the Republic of Buryatia in 2020 (Ivolginsky District, 51°44′48.8040″ N, 107°14′59.4194″ E). To study the seasonal profile of phenolic compounds, samples of *R. matsumuranus* leaves were collected during various growth periods: active growth (15 May), flowering (17 July) and fruiting phases (10 September). All samples were taken in the morning between 10 and 11 a.m. The collected samples were placed in plastic containers and placed in a cooler with ice, where they were kept for several hours during delivery to the laboratory. Leaf samples were dried under laboratory conditions in air for 20 days at a temperature of 24 °C in a ventilated fume hood to a moisture content of 10–14%. The obtained leaf samples were stored at 4 °C before analysis in a Plant Repository of the Institute of General and Experimental Biology. After combining the leaf samples from each harvest date, three total samples of each growth period were obtained. No. Ro/ru-2351-31/15 (May samples), Ro/ru-2352-31/17 (July samples), Ro/ru-2353-31/10 (September samples) were the numbers of voucher specimens of *R. matsumuranus* leaves in the Plant Repository. The samples were ground before analysis in an A11 basic analytical mill (IKA^®^-WerkeGmbh & Co. KG, Staufen, Germany). After grinding, the samples were sieved to a particle size of 0.5 mm on an ERL-M1 sieving machine (Zernotekhnika, Moscow, Russia).

### 3.3. High-Performance Liquid Chromatography with Photodiode Array Detection and Electrospray Ionization Triple Quadrupole Mass Spectrometric Detection (HPLC-PDA-ESI-tQ-MS): Metabolite Profiling and Quantification

Metabolite profiling of *R. matsumuranus* leaves was realized using high-performance liquid chromatography with photodiode array detection and electrospray ionization triple quadrupole mass spectrometric detection (HPLC-PDA-ESI-tQ-MS) performed on a liquid chromatograph LC-20 Prominence coupled photodiode array detector SPD-M30A (wavelength range 200–600 nm), triple-quadrupole mass spectrometer LCMS 8050 (all Shimadzu, Columbia, MD, USA), and C18 column (GLC Mastro; 150 × 2.1 mm, Ø 3 µm; Shimadzu, Kyoto, Japan) at a column temperature of 24 °C. Gradient elution was implemented with two eluents A (0.5% HCOOH in water) and B (0.5% HCOOH in acetonitrile) and the following gradient program: 0–5 min 5–7% B, 5–7 min 7–8% B, 7–10 min 8–19% B, 10–14 min 19–29% B, 14–20 min 29–52% B, 20–25 min 52–73% B, 25–40 min 73–90% B, and 40–50 min 90–5% B. The values of the injection volume and elution flow were 1 µL and 100 µL/min, respectively. The UV-Vis spectra were obtained in the spectral range of 200–600 nm. MS detection was performed in negative ESI mode using the parameters as follows: temperature levels of ESI interface, desolvation line, and heat block were 300 °C, 250 °C, and 400 °C, respectively. The flow levels of nebulizing gas (N_2_), heating gas (air), and collision-induced dissociation gas (Ar) were 3 L/min, 10 L/min, and 0.3 mL/min, respectively. The MS spectra were recorded in the negative mode (−3–−5 kV source voltage) by scanning in the range of *m*/*z* 50–2000 at the collision energy of 5–40 eV. The system was managed under LabSolution’s workstation software with the inner LC-MS library. The identification of compounds was done by the analysis of their retention time, ultraviolet, and mass-spectrometric data, comparing the same parameters with the reference samples and/or literature data.

To quantify compounds **1–63** in *R. matsumuranus* leaves, the reference standards (25 compounds) were accurately weighed (10 mg) and individually dissolved in a DMSO-50% methanol mixture (1:10) in a volumetric flask (10 mL). The stock solutions were used to build external standard calibration curves generated using six data points, 100, 50, 25, 10, 5, and 1 µg/mL, followed by plotting the MS peak area vs. the concentration levels. The validation criteria (correlation coefficients, r^2^; standard deviation, S_YX_; limits of detection, LOD; limits of quantification, LOQ; and linear ranges) were calculated using the previous recommendations [94] (Table S3). All analyses were carried out in triplicate, and the data were expressed as the mean value ± standard deviation (SD). The sample solution was prepared from dried *R. matsumuranus* leaves (50 mg) and 5 mL of methanol in an Eppendorf tube. The mixture was sonicated for 30 min at 50 °C (ultrasound power 100 W, frequency 35 kHz), centrifuged (6000× *g*), filtered (using 0.22-m PTFE syringe filter), and transferred to the volumetric flask (10 mL), and the final volume was reduced to 10 mL by 50% MeOH before HPLC-ESI-tQ-MS analysis.

### 3.4. Methanol Extract Preparation from R. matsumuranus Leaves

Methanol extract of *R. matsumuranus* leaves was prepared as follows: dried crushed leaves (100 g) were extracted twice with stirring in a glass flask (2 L) with methanol (1000 mL). Extraction conditions were an ultrasonic bath, Sapphire 2.8 (Sapphire Ltd., Moscow, Russia), for 30 min and at 50 °C (ultrasound power 100 W and frequency 35 kHz). The resulting extracts were filtered through a cellulose filter, and then the extracts were combined and evaporated in a vacuum until dryness. The obtained methanol extract was kept at 4 °C until further chemical analysis and study of biological activity.

### 3.5. HPLC-PDA-Based Antioxidant Potential: DPPH Scavenging and Fe^2+^-Chelating Activity

Microplate spectrophotometric assay was used to study the scavenging activity of the *R. matsumuranus* methanol extract (1000 µg/mL) against 2,2-diphenyl-1-picrylhydrazyl radicals (DPPH^•^) [26]. The known HPLC-PDA assays coupled with DPPH [25] and FeSO_4_ [78] precolumn incubation were used to study the antioxidant potential of a sample of the *R. matsumuranus* extract (solution in methanol, 1000 µg/mL) in the chromatographic conditions described in Section 3.3. The most active antioxidants showed a strong decrease in the chromatographic area of the separate compounds.

### 3.6. Preparation of R. matsumuranus Leaf Infusions and Decoctions

Infusions and decoctions of *R. matsumuranus* were prepared using the optimal parameters for the extraction of ellagitannin-containing raw materials [27]. To prepare the infusions, accurately weighed leaves (1 g) were mixed with 100 mL of water (20, 50, 80, or 100 °C) in a conical flask and agitated (40 min), followed by cooling at 20 °C (if required), filtering through a PTFE filter (0.45 µm) into a volumetric flask (100 mL), and reducing the final volume with distilled water. The decoctions were produced from accurately weighed leaves (1 g) after mixing with distilled water (100 mL) in a conical flask, heating on a hotplate, and boiling (15 or 30 min). The resultant decoctions were passed through a PTFE filter (0.45 µm) into a volumetric flask (100 mL) and the volume was filled with distilled water. HPLC-PDA-ESI-tQ-MS quantification of the obtained decoctions and infusions without pretreatment was realized in the chromatographic conditions described in Section 3.3. Infusions and decoctions were kept at 4 °C until further chemical analysis.

### 3.7. Statistical Analysis

Statistical analyses were carried out with the usage of ANOVA (one-way analysis of variance). The significance of the mean difference was established by Duncan’s multiple range test. Differences were regarded as statistically considerable at *p* < 0.05. The results are presented as the mean values ± standard deviations (SD).

## 4. Conclusions

*Rubus matsumuranus* is plant species that is widely used by the Eurasian nomads, having a sufficient raw material base. In the present study, the metabolites of *R. matsumuranus* leaves in three stages (active growth, flowering, and fruiting) were studied for the first time. Using the HPLC-PDA-ESI-tQ-MS/MS technique, more than 60 phenolic compounds were identified, particularly gallic acid derivatives, hydroxicinnamates, catechins, procyanidins, flavonols, and ellagitannins. Lambertianin C, miquelianin, and kaempferol-3-*O*-glucuronide were the major compounds of the *R. matsumuranus* leaf phenolome. Due to the abundance of phenolic compounds, the *R. matsumuranus* leaf extract had an antioxidant effect against DPPH^•^ radicals and possessed Fe^2+^-chelating activity. Practical recommendations regarding the time of harvest of *R. matsumuranus* leaves in Siberia were obtained. As a result of the study on seasonal changes in the phenolome, it was revealed that the maximum accumulation of ellagitannins and flavonols occurs in July. To use herbal tea from *R. matsumuranus* leaves, preparing a hot infusion is recommended, as this preparation was enriched with phenolic compounds. Thus, the information presented highlights the potential of *R. matsumuranus* leaves as a source of phenolic compounds that can be included in the human diet to prevent oxidative stress during various diseases.

## Figures and Tables

**Table 1:** Chromatographic (t_R_) and mass-spectrometric data, and seasonal presence/content of compounds **1**–**63** found in *R. matsumuranus* leaves.

No	t_R_, min	Compound * [Ref.]	[M-H]^−^, *m*/*z*	MS/MS, *m*/*z*	Seasonal Content, mg/g DW ** ± SD		
					May (*n* = 32)	July (*n* = 54)	September (*n* = 44)
1	3.22	*O*-Galloyl-dihexose ^L^ [25]	493	331, 169	<0.01 ^b^	0.25 ± 0.00 ^a^	<0.01 ^b^
2	6.82	1-*O*-Caffeoylquinic acid ^S^ [26]	353	191, 179, 173, 135	0.93 ± 0.02 ^a^	0.80 ± 0.02 ^b^	0.56 ± 0.01 ^c^
3	7.18	2-Pyrone-4,6-dicarboxyllic acid ^S^ [27]	183		0.79 ± 0.02 ^c^	2.11 ± 0.04 ^a^	1.83 ± 0.04 ^b^
4	8.49	Gallic acid ^S^ [28]	169		2.03 ± 0.04 ^a^	1.09 ± 0.02 ^b^	0.24 ± 0.00 ^c^
5	8.79	1-*O*-Galloyl-glucose (glucogallin) ^S^ [25]	331	169	<0.01 ^a^	<0.01 ^a^	<0.01 ^a^
6	9.05	Pedunculagin ^S^ [29]	783, 391 ***	633, 481, 301	<0.01 ^c^	0.67 ± 0.02 ^b^	0.92 ± 0.02 ^a^
7	9.73	*O*-Galloyl-hexose ^L^ [25]	331	169	<0.01 ^a^	<0.01 ^a^	<0.01 ^a^
8	10.02	Gallocatechin ^S^ [25]	305	168, 125	2.18 ± 0.04 ^c^	3.57 ± 0.07 ^a^	3.06 ± 0.07 ^b^
9	10.51	Procyanidin B_1_ ^S^ [27]	577	407, 289, 125	1.73 ± 0.04 ^c^	2.03 ± 0.04 ^b^	2.53 ± 0.05 ^a^
10	10.97	Catechin ^S^ [25]	289	247, 191, 123	0.79 ± 0.02 ^c^	1.52 ± 0.03 ^a^	1.01 ± 0.02 ^b^
11	11.28	Procyanidin B_2_ ^S^ [27]	577	407, 289, 125	0.18 ± 0.00 ^c^	0.59 ± 0.02 ^b^	0.79 ± 0.02 ^a^
12	11.53	Epicatechin ^S^ [25]	289	247, 191, 123	3.53 ± 0.07 ^c^	5.39 ± 0.11 ^a^	4.22 ± 0.08 ^b^
13	11.99	3-*O*-Caffeoylquinic acid ^S^ [26]	353	191, 179, 135	1.79 ± 0.03 ^a^	1.22 ± 0.02 ^b^	0.93 ± 0.02 ^c^
14	12.53	*O*-Caffeoyl-hexose ^L^ [26]	341	179	4.38 ± 0.09 ^a^	3.27 ± 0.06 ^b^	1.40 ± 0.02 ^c^
15	13.02	Tellimagrandin I_1_ ^S^ [27]	785, 392 ***	633, 483, 301	0.93 ± 0.02 ^b^	1.93 ± 0.04 ^a^	0.38 ± 0.00 ^c^
16	13.09	1,6-Di-*O*-galloyl-glucose ^S^ [25]	483	331, 169	1.18 ± 0.02 ^a^	0.33 ± 0.02 ^c^	0.52 ± 0.01 ^b^
17	14.35	5-*O*-Caffeoylquinic acid ^S^ [26]	353	191, 179, 165	3.53 ± 0.07 ^a^	2.81 ± 0.05 ^b^	2.03 ± 0.04 ^c^
18	14.48	4-*O*-Caffeoylquinic acid ^S^ [26]	353	191, 179, 173, 135	2.36 ± 0.04 ^a^	1.73 ± 0.03 ^b^	1.48 ± 0.03 ^c^
19	14.73	5-*O*-Feruloylquinic acid ^S^ [26]	367	205, 193	3.86 ± 0.07 ^a^	3.07 ± 0.05 ^b^	1.98 ± 0.04 ^c^
20	15.09	Tellimagrandin I_2_ ^S^ [27]	785, 392 ***	633, 483, 301	0.26 ± 0.00 ^c^	0.93 ± 0.02 ^a^	0.31 ± 0.00 ^b^
21	15.46	Tellimagrandin II_1_ ^S^ [27]	937	785, 767,599,465, 301	0.12 ± 0.00 ^b^	0.38 ± 0.00 ^a^	<0.01 ^c^
22	15.92	Potentillin ^S^ [27]	935, 467 ***	633, 463, 301	0.45 ± 0.01 ^c^	1.63 ± 0.03 ^a^	1.28 ± 0.02 ^b^
23	16.03	1,3,6,-Tri-*O*-galloyl-glucose ^S^ [25]	635	483, 331, 169, 125	2.90 ± 0.06 ^a^	1.67 ± 0.03 ^c^	1.93 ± 0.04 ^b^
24	16.51	Tri-*O*-galloyl-hexose ^L^ [25]	635	483, 331, 169, 125	2.27 ± 0.04 ^a^	1.26 ± 0.03 ^c^	1.48 ± 0.03 ^b^
25	16.77	Sanguiin H10 ^L^ [29]	1567,783 ***	933, 633, 301	0.93 ± 0.02 ^b^	1.91 ± 0.04 ^a^	0.67 ± 0.02 ^c^
26	17.41	Lambertianin A ^S^ [29]	1869,934 ***	1265,935,783,633,481,301	1.83 ± 0.03 ^c^	5.21 ± 0.11 ^a^	4.63 ± 0.09 ^b^
27	18.21	Pedunculagin isomer ^L^ [29]	783	481, 301	<0.01 ^c^	0.14 ± 0.00 ^b^	0.44 ± 0.01 ^a^
28	18.48	Tellimagrandin II_2_ ^S^ [27]	937	785, 767,599,465, 301	0.07 ± 0.00 ^b^	0.36 ± 0.00 ^a^	<0.01 ^c^
29	18.63	Sanguiin H6 ^S^ [29]	1567,783 ***	933, 633, 301	6.14 ± 0.14 ^c^	19.62 ± 0.39 ^a^	15.32 ± 0.31 ^b^
30	19.14	Lambertianin C ^S^ [29]	1401	783, 633, 301	25.18 ± 0.50 ^c^	57.11 ± 1.14 ^a^	48.10 ± 0.96 ^b^
31	20.45	Catechin *O*-gallate ^S^ [25]	441	289, 125, 109	0.36 ± 0.00 ^c^	1.63 ± 0.03 ^b^	1.58 ± 0.03 ^a^
32	20.81	Ellagic acid *O*-pentoside-*O*-hexoside ^L^ [27]	595	433, 301	0.27 ± 0.00 ^c^	1.35 ± 0.02 ^a^	0.93 ± 0.02 ^b^
33	21.46	Sanguiin H11 ^S^ [27]	951	799, 481, 301	0.14 ± 0.00 ^c^	2.03 ± 0.04 ^a^	1.27 ± 0.03 ^b^
34	21.78	Ellagic acid *O*-hexoside ^L^ [27]	463	301	<0.01 ^b^	0.52 ± 0.02 ^a^	<0.01 ^b^
35	22.02	Ellagic acid *O*-hexoside ^L^ [27]	463	301	<0.01 ^b^	0.40 ± 0.01 ^a^	<0.01 ^b^
36	22.71	Ellagic acid *O*-pentoside ^L^ [27]	433	301	<0.01 ^b^	0.49 ± 0.01 ^a^	<0.01 ^b^
37	23.00	Ellagic acid ^S^ [27]	301		1.67 ± 0.03 ^c^	6.24 ± 0.12 ^b^	11.20 ± 0.23 ^a^
38	23.43	Quercetin-3-*O*-rutinoside (rutin) ^S^ [26,27,29]	609	463, 301	0.09 ± 0.00 ^c^	0.96 ± 0.02 ^a^	0.11 ± 0.00 ^b^
39	24.69	Quercetin-3-*O*-glucoside (isoquercitrin) ^S^ [26,27,29]	463	301	<0.01 ^b^	0.52 ± 0.01 ^a^	<0.01 ^b^
40	24.42	Quercetin-3-*O*-glucuronide (miquelianin) ^S^ [26,27,29]	477	301	14.22 ± 0.29 ^c^	39.63 ± 0.78 ^a^	31.15 ± 0.63 ^b^
41	25.11	Kaempferol-3-*O*-glucuronide ^S^ [26,27,29]	461	285	9.23 ± 0.18 ^c^	31.18 ± 0.60 ^a^	25.67 ± 0.51 ^b^
42	25.44	Quercetin *O*-(*O*-malonyl)-hexuronide ^L^ [26,27,29]	563	477, 301	0.63 ± 0.02 ^b^	2.61 ± 0.05 ^a^	0.57 ± 0.01 ^c^
43	25.69	Kaempferol *O*-(*O*-malonyl)-hexuronide ^L^ [26,27,29]	533	447, 285	<0.01 ^a^	<0.01 ^a^	<0.01 ^a^
44	26.39	Quercetin *O*-(*O*-acetyl)-hexuronide ^L^ [26,27,29]	519	477, 301	0.18 ± 0.00 ^c^	2.20 ± 0.04 ^a^	0.20 ± 0.00 ^b^
45	26.81	Kaempferol *O*-(*O*-acetyl)-hexuronide ^L^ [26,27,29]	503	461, 285	<0.01 ^a^	<0.01 ^a^	<0.01 ^a^
46	27.31	Quercetin *O*-(*O*-acetyl-*O*-malonyl)-hexuronide ^L^	605	519, 477, 301	18.69 ± 0.36 ^c^	36.82 ± 0.73 ^a^	21.03 ± 0.42 ^b^
47	27.93	Quercetin *O*-(*O*-acetyl-*O*-malonyl)-hexuronide ^L^	605	519, 477, 301	<0.01 ^a^	<0.01 ^a^	<0.01 ^a^
48	28.63	Kaempferol *O*-(*O*-acetyl-*O*-malonyl)-hexuronide ^L^	589	503, 461, 285	0.04 ± 0.00 ^c^	1.83 ± 0.04 ^a^	0.30 ± 0.00 ^b^
49	29.47	Kaempferol *O*-(*O*-acetyl-*O*-malonyl)-hexuronide ^L^	589	503, 461, 285	<0.01 ^b^	0.31 ± 0.00 ^a^	<0.01 ^b^
50	29.83	Quercetin *O*-(*O*-acetyl-di-*O*-malonyl)-hexuronide ^L^	691	605, 519, 477, 301	<0.01 ^a^	<0.01 ^a^	<0.01 ^a^
51	30.33	Kaempferol *O*-(*O*-acetyl-di-*O*-malonyl)-hexuronide ^L^	675	589, 503, 461, 285	<0.01 ^b^	0.26 ± 0.00 ^a^	<0.01 ^b^
52	31.06	Quercetin *O*-(di-*O*-acetyl-*O*-malonyl)-hexuronide ^L^	647	561, 519, 477, 301	<0.01 ^a^	<0.01 ^a^	<0.01 ^a^
53	31.27	Kaempferol *O*-(di-*O*-acetyl-*O*-malonyl)-hexuronide ^L^	631	545, 503, 461, 285	<0.01 ^a^	<0.01 ^a^	<0.01 ^a^
54	31.90	Ellagic acid *O*-methyl ester *O*-pentoside ^L^ [26,29]	447	315, 301	<0.01 ^a^	<0.01 ^a^	<0.01 ^a^
55	32.41	Ellagic acid *O*-methyl ester *O*-pentoside ^L^ [26,29]	447	315, 301	<0.01 ^a^	<0.01 ^a^	<0.01 ^a^
56	32.58	Quercetin *O*-(tri-*O*-acetyl)-hexuronide ^L^ [26,29]	603	561, 519, 477, 301	<0.01 ^c^	0.82 ± 0.02 ^a^	0.22 ± 0.00 ^b^
57	32.79	Quercetin *O*-(tri-*O*-acetyl)-hexuronide ^L^ [26,29]	603	561, 519, 477, 301	<0.01 ^b^	0.08 ± 0.00 ^a^	<0.01 ^b^
58	33.60	Quercetin *O*-(tri-*O*-acetyl-*O*-malonyl)-hexuronide ^L^	689	603, 561, 519, 477, 301	<0.01 ^b^	<0.01 ^b^	0.10 ± 0.00 ^a^
59	34.01	Kaempferol *O*-(tri-*O*-acetyl)-hexuronide ^L^ [26,29]	587	545, 503, 461, 285	<0.01 ^a^	<0.01 ^a^	<0.01 ^a^
60	34.99	Kaempferol *O*-(tri-*O*-acetyl-*O*-malonyl)-hexuronide ^L^	673	587, 545, 503, 461, 285	<0.01 ^a^	<0.01 ^a^	<0.01 ^a^
61	35.72	Ellagic acid *O*-di-methyl ester ^L^ [26,29]	329	315, 301	<0.01 ^a^	<0.01 ^a^	<0.01 ^a^
62	36.53	Ellagic acid *O*-di-methyl ester ^L^ [26,29]	329	315, 301	<0.01 ^a^	<0.01 ^a^	<0.01 ^a^
63	38.11	Ellagic acid *O*-tri-methyl ester ^L^ [26,29]	343	329, 315, 301	<0.01 ^a^	<0.01 ^a^	<0.01 ^a^

* Compound identification was based on comparison of the retention time, UV and MS spectral data with the reference standard (^S^), or interpretation of UV and MS spectral data and comparison with the literature data (^L^). ** Content in *R. matsumuranus* leaves collected in various months (from May to September). ***—additional ion [M–2H]^2–^. *n*—number of plant samples used for analysis. DW—dry plant weight. Values with different letters (a–c) indicate statistically significant differences among groups at *p* < 0.05 by one-way ANOVA.

**Table 2:** Phenolic content in *R. matsumuranus* tea infusions and decoctions (mg/100 mL ± SD).

Compound	Cold Infusion (20 °C)	Warm Infusion (50 °C)	Hot Infusion (80 °C)	Boiling Infusion (100 °C)	Decoction 15 min	Decoction 30 min
Ellagic acid and Ellagitannins						
Ellagic acid	1.27 ± 0.03 ^f^	1.54 ± 0.03 ^e^	1.96 ± 0.05 ^d^	3.39 ± 0.07 ^c^	15.89 ± 0.37 ^b^	18.25 ± 0.37 ^a^
Lambertianin A	1.11 ± 0.02 ^c^	1.68 ± 0.03 ^a^	1.35 ± 0.03 ^b^	0.21 ± 0.01 ^d^	<0.01 ^e^	<0.01 ^e^
Sanguiin H6	5.25 ± 0.10 ^c^	6.13 ± 0.12 ^a^	5.40 ± 0.11 ^b^	4.01 ± 0.08 ^d^	1.11 ± 0.07 ^e^	<0.01 ^f^
Lambertianin C	13.05 ± 0.27 ^c^	16.32 ± 0.30 ^a^	14.96 ± 0.30 ^b^	9.03 ± 0.19 ^d^	2.12 ± 0.11 ^e^	<0.01 ^f^
Sanguiin H11	0.19 ± 0.00 ^c^	0.51 ± 0.01 ^a^	0.36 ± 0.01 ^b^	<0.01 ^d^	<0.01 ^d^	<0.01 ^d^
Subtotal ellagic acid and ellagitannins	20.87	26.18	24.03	16.64	19.12	18.25
Catechins						
Gallocatechin	0.19 ± 0.00 ^f^	0.35 ± 0.01 ^e^	0.60 ± 0.01 ^d^	0.77 ± 0.02 ^c^	0.92 ± 0.02 ^a^	0.85 ± 0.02 ^b^
Epicatechin	0.26 ± 0.01 ^f^	0.40 ± 0.01 ^e^	0.72 ± 0.02 ^d^	0.93 ± 0.02 ^c^	1.02 ± 0.02 ^b^	1.17 ± 0.02 ^a^
Subtotal catechins	0.45	0.75	1.32	1.70	1.94	2.02
Hydroxycinnamates						
*O*-Caffeoyl-hexose	0.33 ± 0.01 ^f^	0.52 ± 0.01 ^e^	0.86 ± 0.02 ^d^	1.06 ± 0.03 ^a^	1.01 ± 0.03 ^b^	0.93 ± 0.02 ^c^
4-*O*-Caffeoyquinic acid	<0.01 ^f^	0.22 ± 0.00 ^e^	0.58 ± 0.01 ^c^	0.83 ± 0.02 ^a^	0.70 ± 0.01 ^b^	0.55 ± 0.01 ^d^
5-*O*-Caffeoylquinic acid	0.35 ± 0.01 ^f^	0.60 ± 0.01 ^e^	0.88 ± 0.02 ^d^	1.14 ± 0.03 ^a^	1.07 ± 0.03 ^b^	0.96 ± 0.02 ^c^
5-*O*-Feruloylquinic acid	0.29 ± 0.01 ^f^	0.52 ± 0.01 ^e^	0.71 ± 0.02 ^d^	0.94 ± 0.02 ^a^	0.88 ± 0.02 ^b^	0.76 ± 0.02 ^c^
Subtotal hydroxycinnamates	0.97	1.86	3.03	3.97	3.66	3.20
Flavonols						
Quercetin-3-*O*-glucuronide	5.12 ± 0.10 ^e^	6.29 ± 0.14 ^d^	7.02 ± 0.15 ^c^	7.43 ± 0.16 ^b^	8.07 ± 0.16 ^a^	8.21 ± 0.17 ^a^
Quercetin-*O*-(*O*-acetyl-*O*-malonyl)-hexuronide	7.32 ± 0.15 ^d^	8.61 ± 0.17 ^b^	9.14 ± 0.21 ^a^	7.69 ± 0.16 ^c^	5.07 ± 0.10 ^e^	4.21 ± 0.08 ^f^
Kaempferol-3-*O*-glucuronide	3.17 ± 0.06 ^e^	5.08 ± 0.11 ^d^	7.39 ± 0.13 ^c^	8.23 ± 0.16 ^b^	8.51 ± 0.17 ^a^^,b^	8.66 ± 0.18 ^a^
Subtotal flavonols	15.61	19.98	23.55	23.35	21.65	21.08
Total phenolics	37.90	48.77	51.93	45.66	46.37	44.55

Values with different letters (a–f) indicate statistically significant differences among groups at *p* < 0.05 by one-way ANOVA.

## Data Availability

Data is contained within the article.

## Supplementary Materials

The following are available online at https://www.mdpi.com/article/10.3390/plants10112317/s1, Table S1, Total phenolic content in solvents after various types of extraction of *Rubus matsumuranus* leaves; Table S2, Reference standards used for the qualitative and quantitative analysis by HPLC-PDA-ESI-QQQ-MS assay; Table S3, Regression equations, correlation coefficients, standard deviation, limits of detection, limits of quantification and linear ranges for 25 reference standards.
