# Supplementary material for: Metabolites of Siberian Raspberries: LC-MS Profile, Seasonal Variation, Antioxidant Activity and, Thermal Stability of Rubus matsumuranus Phenolome
Source: Plants (Basel). 2021 Oct 27;10(11):2317. doi: 10.3390/plants10112317 (PMC8620613; doi:10.3390/plants10112317)
Supplement: Supplementary file 1 [file plants-10-02317-s001.zip › plants-1410158-supplementary.pdf]

plants
MDPI

Supplementary materials

Metabolites of Siberian Raspberries: LC-MS Profile, Seasonal Variation, Antioxidant Activity and Thermal Stability of *Rubus matsumuranus* Phenolome

Nina I. Kashchenko[1*], Daniil N. Olennikov[1] and Nadezhda K. Chirikova[2]

[1] Laboratory of Medical and Biological Research, Institute of General and Experimental Biology, Siberian Division, Russian Academy of Science, 670047 Ulan-Ude, Russia; olennikovdn@mail.ru (D.N.O.)
[2] Department of Biology, Institute of Natural Sciences, North-Eastern Federal University, 677027 Yakutsk, Russia; hofnung@mail.ru (N.K.C.)
* Correspondence: ninkk@mail.ru; Tel.: +7-9834-217-340

Content

Table S1. Total phenolic content in solvents after various types of extraction of *Rubus matsumuranus* leaves, μg/mL ± S.D.

Table S2. Reference standards used for the qualitative and quantitative analysis by HPLC-PDA-ESI-QQQ-MS assay

Table S3. Regression equations, correlation coefficients (r^2), standard deviation (S_{YX}), limits of detection (LOD), limits of quantification (LOQ) and linear ranges for 25 reference standards.

Table S1. Total phenolic content in solvents after various types of extraction of *Rubus matsumuranus* leaves, µg/mL ± S.D.

Parameter	TPC	Parameter	TPC
Solvent		Solvent-material ratio	
Methanol	2.25 ± 0.05	1 : 2	1.67 ± 0.03
Ethanol	2.11 ± 0.04	1 : 5	1.98 ± 0.04
Isopropanol	1.77 ± 0.04	1 : 10	2.29 ± 0.05
Water	1.12 ± 0.02	1 : 20	2.27 ± 0.05
Extraction type *		Temperature regime	
RTE	1.17 ± 0.02	10°C	1.15 ± 0.02
BWBE	1.96 ± 0.04	30°C	1.33 ± 0.03
MWAE	2.08 ± 0.05	50°C	2.23 ± 0.04
USE	2.30 ± 0.05	70°C	2.17 ± 0.05
		90°C	1.65 ± 0.03

* Extraction type: RTE – room temperature extraction (20°C), BWBE – boiled water bath extraction (95°C), MWAE – microwave-assisted extraction (20°C), USE – ultrasound extraction (50°C). Total phenolic content was calculated as a sum of compounds **1**–**63** (Table 1) after HPLC quantification (Material and Methods, Section 3.3).

Table S2. Reference standards used for the qualitative and quantitative analysis by HPLC-PDA-ESI-QQQ-MS assay.

No	Compound	Purity (≥), %	Manufacturer (Cat. No.) *	Used for analysis (compound No in Table 1)
**2**	1-*O*-Caffeoylquinic acid	98	ChemFaces (CFN99121)	**2**
**4**	Gallic acid	98	Sigma (PHL89198)	**4**
**5**	Glucogallin	90	Sigma (PHL83250)	**1, 5, 7**
**6**	Pedunculagin	95	Toronto (P354070)	**6, 25, 26, 27, 29, 30, 33**
**8**	Gallocatechin	97	Sigma (01388)	**8**
**9**	Procyanidin B_1	90	Sigma (19542)	**9**
**10**	Catechin	99	Sigma (43412)	**10**
**11**	Procyanidin B_2	90	Sigma (42157)	**11**
**12**	Epicatechin	97	Sigma (68097)	**12**
**13**	3-*O*-Caffeoylquinic acid	95	Sigma (PHL89175)	**13**
**16**	1,6-Di-*O*-galloyl-glucose	92	Toronto (D293195)	**16**
**17**	5-*O*-Caffeoylquinic acid	95	Sigma (91213)	**17**
**18**	4-*O*-Caffeoylquinic acid	98	Sigma (65969)	**18**
**19**	5-*O*-Feruloylquinic acid	98	ChemFaces (CFN92889)	**19**
**23**	1,3,6-Tri-*O*-galloyl-glucose	98	Sigma (78864)	**23, 24**
**31**	Catechin *O*-gallate	98	Sigma (C0692)	**31**
**37**	Ellagic acid	98	Sigma (PHL89653)	**32, 34, 35, 36, 37, 54, 55, 61, 62, 63**
**38**	Quercetin-3-*O*-rutinoside	95	Sigma (PHL89270)	**38**
**39**	Quercetin-3-*O*-glucoside	95	Sigma (00140585)	**39**
**40**	Quercetin-3-*O*-glucuronide	90	Sigma (90733)	**40, 42, 44, 46, 47, 50, 52, 56, 57, 58**
**41**	Kaempferol-3-*O*-glucuronide	97	Sigma (79273)	**41, 43, 45, 48, 49, 51, 53, 59, 60**
	Coumalic acid	97	Sigma (C85409)	**3**
	Caffeic acid	98	Sigma (C0625)	**14**
	Agrimoniin	98	ALB (82203-01-8)	**22**
	Corilagin	98	Sigma (G0424)	**15, 20, 21, 28**

* Manufacturers list: ALB (ALB Technology Limited, Mongkok Kowloon HongKong); ChemFaces (Wuhan, Hubei, PRC); Sigma-Aldrich (St. Louis, MO, USA); Toronto (Toronto Research Chemicals, North York, ON, Canada);

Table S3. Regression equations, correlation coefficients (r^2), standard deviation (S_{YX}), limits of detection (LOD), limits of quantification (LOQ) and linear ranges for 25 reference standards.

Compound	Ionization [a]	CE [b] (eV)	Regression equation [c]		r^2	S_{yx}	LOD/LOQ (µg/mL)	Linear range (µg/mL)
			a	$b \cdot 10^6$				
1-*O*-Caffeoylquinic acid	N	-15	2.5394	-1.2360	0.9994	$0.45 \cdot 10^{-2}$	0.006/0.02	0.02–300.0
Gallic acid	N	-20	2.6538	-0.1376	0.9990	$1.17 \cdot 10^{-2}$	0.01/0.04	0.1–100.0
Glucogallin	N	-20	1.3586	-0.0663	0.9987	$9.69 \cdot 10^{-2}$	0.24/0.71	0.8–100.0
Pedunculagin	N	-35	0.6370	-0.4521	0.9872	$6.11 \cdot 10^{-2}$	0.32/0.96	1.0–100.0
Gallocatechin	N	-35	1.1495	-0.2110	0.9982	$17.02 \cdot 10^{-2}$	0.48/1.48	1.5–100.0
Procyanidin B_1	N	-30	1.3722	-0.0829	0.9973	$9.93 \cdot 10^{-2}$	0.24/0.72	0.80–100.0
Catechin	N	-35	0.9562	-0.0521	0.9971	$7.79 \cdot 10^{-2}$	0.27/0.82	0.9–100.0
Procyanidin B_2	N	-25	1.3620	-0.0820	0.9961	$9.91 \cdot 10^{-2}$	0.21/0.72	0.8–100.0
Epicatechin	N	-35	1.0828	-0.0456	0.9973	$6.85 \cdot 10^{-2}$	0.21/0.63	0.7–100.0
3-*O*-Caffeoylquinic acid	N	-15	0.9320	-0.0523	0.9991	$4.14 \cdot 10^{-2}$	0.15/0.44	0.5–100.0
1,6-Di-*O*-galloyl-glucose	N	-20	1.7552	-0.0569	0.9982	$8.89 \cdot 10^{-2}$	0.18/0.51	0.6–100.0
5-*O*-Caffeoylquinic acid	N	-15	0.9406	-0.0497	0.9973	$5.18 \cdot 10^{-2}$	0.18/0.55	0.6–100.0
4-*O*-Caffeoylquinic acid	N	-15	0.9217	-0.0437	0.9982	$3.94 \cdot 10^{-2}$	0.14/0.43	0.5–100.0
5-*O*-Feruloylquinic acid	N	-20	1.8535	0.0761	0.9989	$4.55 \cdot 10^{-2}$	0.08/0.25	0.3–100.0
1,3,6-Tri-*O*-galloyl-glucose	N	-25	2.1064	-0.0499	0.9985	$8.74 \cdot 10^{-2}$	0.14/0.42	0.5–100.0
Catechin *O*-gallate	N	-35	1.3387	-0.0284	0.9981	$9.50 \cdot 10^{-2}$	0.23/0.71	0.8–100.0
Ellagic acid	N	-30	0.9114	-0.6312	0.9887	$6.37 \cdot 10^{-2}$	0.23/0.70	0.7–100.0
Quercetin-3-*O*-rutinoside	N	-25	1.2716	-0.7389	0.9897	$9.14 \cdot 10^{-2}$	0.23/0.72	0.8–100.0
Quercetin-3-*O*-glucoside	N	-20	1.8267	-0.4160	0.9990	$11.73 \cdot 10^{-2}$	0.21/0.67	0.7–100.0
Quercetin-3-*O*-glucuronide	N	-20	1.6705	-0.4374	0.9988	$12.79 \cdot 10^{-2}$	0.25/0.77	0.8–100.0
Kaempferol-3-*O*-glucuronide	N	-30	2.2126	-0.5160	0.9987	$8.11 \cdot 10^{-2}$	0.12/0.37	0.4–100.0
Coumalic acid	N	-20	0.8115	-0.1006	0.9980	$2.25 \cdot 10^{-2}$	0.10/0.28	0.3–100.0
Caffeic acid	N	-20	2.4493	-0.0938	0.9989	$1.85 \cdot 10^{-2}$	0.03/0.08	0.1–100.0
Agrimoniin	N	-35	0.8214	-0.2716	0.9893	$5.37 \cdot 10^{-2}$	0.22/0.65	0.7–100.0
Corilagin	N	-35	0.9361	-0.4518	0.9870	$9.35 \cdot 10^{-2}$	0.32/1.00	1.0–100.0

[a] Ionization mode: N – negative. [b] CE – collision energy. [c] Regression equation: $y = a \cdot x + b$
